# Electrochemically Stable Rechargeable Lithium–Sulfur Batteries Equipped with an Electrospun Polyacrylonitrile Nanofiber Film

**DOI:** 10.3390/polym15061460

**Published:** 2023-03-15

**Authors:** Li-Ling Chiu, Sheng-Heng Chung

**Affiliations:** 1 Department of Materials Science and Engineering, National Cheng Kung University, No. 1, University Road, Tainan City 70101, Taiwan; 2 Hierarchical Green-Energy Materials Research Center, National Cheng Kung University, No. 1, University Road, Tainan City 70101, Taiwan

**Keywords:** electrospinning, high-loading cathodes, stabilized lithium anodes, energy density, lithium–sulfur batteries

## Abstract

The high theoretical charge-storage capacity and energy density of lithium–sulfur batteries make them a promising next-generation energy-storage system. However, liquid polysulfides are highly soluble in the electrolytes used in lithium–sulfur batteries, which results in irreversible loss of their active materials and rapid capacity degradation. In this study, we adopt the widely applied electrospinning method to fabricate an electrospun polyacrylonitrile film containing non-nanoporous fibers bearing continuous electrolyte tunnels and demonstrate that this serves as an effective separator in lithium–sulfur batteries. This polyacrylonitrile film exhibits high mechanical strength and supports a stable lithium stripping and plating reaction that persists for 1000 h, thereby protecting a lithium-metal electrode. The polyacrylonitrile film also enables a polysulfide cathode to attain high sulfur loadings (4–16 mg cm^−2^) and superior performance from C/20 to 1C with a long cycle life (200 cycles). The high reaction capability and stability of the polysulfide cathode result from the high polysulfide retention and smooth lithium-ion diffusion of the polyacrylonitrile film, which endows the lithium–sulfur cells with high areal capacities (7.0–8.6 mA·h cm^−2^) and energy densities (14.7–18.1 mW·h cm^−2^).

## 1. Introduction

Lithium-ion battery technology is mature and approaching the theoretical limits of the charge-storage capacities and energy densities of the active materials. Thus, in addition to the studies focused on optimizing the active material, device configuration, and fabrication method of lithium-ion batteries, there are increasing efforts to develop novel energy-storage systems with next-generation electrode materials featuring either high operating voltages or high charge-storage capacities. Next-generation electrode materials are needed to meet the requirements of the current electrical devices for batteries that have high energy densities, low toxicity, and low cost [1,2,3]. Among the next-generation electrode materials, lithium–sulfur batteries use a non-toxic sulfur cathode, which has a high theoretical discharge capacity and energy density (1672 mA·h g^−1^ and 2500 W·h kg^−1^, respectively) via the redox reaction of Li ⇄ Li^+^ + e^-^ and S + 2 Li^+^ + 2e^-^ ⇄ Li_2_S (E° ≈ 2.15 V vs. Li/Li^+^). Accordingly, the rechargeable lithium–sulfur battery offers 10 times the capacity and 3–5 times the energy density of current rechargeable lithium-ion batteries [4,5,6]. This is because, unlike the insertion reaction that occurs on lithium-ion battery cathodes, the reaction on sulfur cathodes is an electrochemical conversion reaction that allows the full and reversible utilization of two electrons per sulfur via the redox reaction of sulfur and lithium sulfide, which generates a high charge-storage capacity [6,7,8,9]. The superior electrochemical characteristics of lithium–sulfur batteries and the high abundance and wide distribution of sulfur mean that such batteries would be effective and cost-efficient [10,11].

However, despite the above-mentioned electrochemical and device advantages of lithium–sulfur cells, the low conductivity and poor stability of sulfur cathodes need to be ameliorated. The low conductivity of their active material in its solid state and their high solubility in its liquid state result in a sluggish reaction and a high loss of liquid active material. Specifically, the electrochemical utilization and reversibility of a lithium–sulfur cell are limited by the insulating nature of sulfur in the charged state and lithium sulfide in the discharged state, which lead to cells having high resistance in these states (1 × 10^−30^ and 1 × 10^−14^ S cm^−1^, respectively) [6,12,13]. To solve this problem, studies have fabricated various composite sulfur cathodes with carbon [14,15,16], metals [17,18,19], ceramics [20,21,22], or conductive polymers [23,24,25] as host materials, which have better electron and/or ion transfer capabilities compared to conventional sulfur cathodes and, thus, have exhibited high charge-storage capacities and high-rate performances [14,15,16,17,18,19,20,21,22,23,24,25]. The performance of lithium–sulfur batteries is also deleteriously affected by polysulfides, which are liquid intermediates that readily dissolve and diffuse in the electrolytes. The polysulfide diffusion causes unstable discharge–charge reactions and an irreversible capacity loss [26,27]. To solve this problem, composite sulfur cathodes have been combined with state-of-the-art interlayer or composite separators to serve as polysulfide-trapping free-standing films or functional coatings on conventional separators, respectively [6,10,11,12,13]. Analogous to the design changes made to the cathode region of lithium–sulfur batteries, the next step is to develop functional separators capable of maintaining the fast diffusion of lithium ions while blocking the uncontrollable passage of polysulfides in lithium–sulfur cells. This is a non-trivial challenge, as additional material characteristics—electrolyte affinity, mechanical strength, and chemical structure—must be considered when developing a lithium–sulfur battery separator [28,29,30,31,32].

In this work, we adopted electrospinning—which is widely used to prepare polymeric fiber films and has been used to make lithium-ion battery separators consisting of polyacrylonitrile [33,34], polymethyl methacrylate [35,36], and polyvinylidene difluoride [37,38]—to fabricate a polyacrylonitrile nanofiber film for use as a separator in lithium–sulfur batteries. This polyacrylonitrile film contained an interconnected lithium-ion transfer network, and exhibited high polysulfide inhibition on the cathode side (i.e., long-term cycle stability: 200 cycles at C/20–1C) and good lithium-dendrite prevention on the anode side (i.e., a long life: 1000 h in lithium-metal cells at 1 mA cm^−2^). Most importantly, we applied the polyacrylonitrile film directly to a high-loading polysulfide cathode with a high sulfur content and loading (52–80 wt% based on the total mass of cathode and 4–16 mg cm^−2^ based on a 1 × 1 cm^2^ electrode, respectively) to demonstrate the designed configuration enabling the high electrochemical utilization and stability of a large amount of polysulfides, which overcomes chemical and engineering problems [8,39,40]. The resulting high-loading polysulfide cathode attained high specific areal capacities (4.1–8.6 mA·h cm^−2^) and energy densities (8.6–18.1 mW·h cm^−2^), which meant that the cell performance of a lithium–sulfur battery containing the polyacrylonitrile film is comparable to or higher than those of commercially available lithium-ion batteries, such as the areal capacity for electric vehicle batteries (2–4 mA·h cm^−2^) and the energy densities of lithium-ion batteries (10–15 mW·h cm^−2^) [6,7,8,9,10,11,12,37,38,39,40]. Our experimental and analytical results confirm that the polyacrylonitrile film can provide excellent electrolyte affinity, good polysulfide inhibition, and smooth lithium stripping/plating reactivity, thereby facilitating the development of a high-loading polysulfide cathode with a high specific capacity, energy density, and stability.

## 2. Materials and Methods

### 2.1. Electrospinning of Polyacrylonitrile Nanofiber Film

The electrospinning solution was prepared by dissolving polyacrylonitrile (average Mw = 150,000 g mol^−1^, Sigma-Aldrich, Darmstadt, Germany) in *N*,*N*-dimethylformamide (DMF, Sigma-Aldrich) to form an 11 wt% solution. The electrospinning was conducted at an applied voltage of 18 kV and a flow rate of 2 mL h^−1^ as the optimal fabrication condition for the preparation of a smooth polyacrylonitrile film with a low number of beaded fibers. The electrospun polyacrylonitrile film was dried at 50 °C for one day and cut into a disk of 19 mm in diameter and 200 µm in thickness, which was used as a lithium–sulfur battery separator.

### 2.2. Material Characterization of Electrospun Polyacrylonitrile Nanofiber Film

The microstructure and morphology of the electrospun polyacrylonitrile nanofiber film before and after cycling were observed by field-emission scanning electron microscopy (SEM, JSM-7001, JEOL, Tokyo, Japan). The polyacrylonitrile film was subjected to Fourier-transform infrared spectrometry (FTIR, Thermo, Nicolet, Green Bay, WI, USA) to determine its chemical structure, gas sorption analysis (Autosorb iQ, Anton Paar, Graz, Austria) to determine its gas adsorption–desorption behavior, microtensile testing (DDS32, Kammrath & Weiss GmbH, Schwerte, Germany) to determine its mechanical strength, and ^7^Li nuclear magnetic resonance (NMR) spectrometry (AV-500, Bruker, Billerica, MA, USA) to determine its electrolyte affinity. The ^7^Li NMR data were used to calculate and fit the diffusion coefficient (*D*), according to: *I*/*I_0_* = exp(-*Dg*^2^*δ*^2^*σ*^2^*g*^2^*Δ*′), where *I* and *I_0_* are the intensity and initial intensity of the selected resonance, respectively; *Δ*′ is the diffusion delay; *δ* is the gradient pulse length; *g* is the gradient pulse strength; and *σ* is the gradient shape factor [41]. The electrolyte uptake and electrolyte retention were calculated by: (*W*_1_ − *W*_0_)/*W*_0_ × 100% and (*W_x_* − *W*_0_)/(*W*_1_ − *W*_0_) × 100%, respectively, where *W*_1_ is the weight of the wetting separators, *W*_0_ is the weight of the origin separators, and *W*_x_ is the weight of the wetting separators after drying for an hour [42,43].

### 2.3. Electrochemical Performance of Electrospun Polyacrylonitrile Nanofiber Film

The polyacrylonitrile film was used as a separator in a lithium//lithium symmetrical cell and a lithium–sulfur electrochemical cell, respectively, and the performance of these cells was analyzed. The performance of the symmetrical cells was examined at a current density of 1 mA cm^−2^ to analyze how the polyacrylonitrile film supported the lithium stripping/plating reaction and protected the lithium electrodes. The lithium–sulfur cells contained a lithium counter/reference electrode and a polysulfide cathode to enable the direct analysis of polysulfide inhibition and retention. The catholyte comprised 1.5 M polysulfide that was fabricated by dissolving sulfur (5 mmol, 99.5%, Alfa Aesar, Haverhill, MA, USA) and lithium sulfide (1 mmol, 99.9%, Alfa Aesar, Haverhill, MA, USA) in a blank electrolyte. The blank electrolyte was composed of 1.85 M lithium bis(trifluoromethanesulfonyl)imide and 0.5 M lithium nitrate (99.98%, Alfa Aesar, Haverhill, MA, USA) dissolved in 1,2-dimethoxyethane (55 vol%, 99+%, Alfa Aesar, Haverhill, MA, USA) and 1,3-dioxolane (40 vol%, 99.5%, Alfa Aesar, Haverhill, MA, USA). This catholyte was dropped onto the current collector to form cells with high sulfur loadings and high sulfur contents (4, 8, 12, and 16 mg cm^−2^ and 52, 68, 75, and 80 wt%, respectively) in the consideration of the total mass of the cathode. A reference cell with a conventional polypropylene separator (19 mm in diameter and 25 µm in thickness) was fabricated at the same high sulfur loadings (4, 8, 12, and 16 mg cm^−2^), and only the cell with a sulfur loading of 4 mg cm^−2^ afforded sufficient experimental data.

The assembled lithium–sulfur cells were analyzed at room temperature (25 °C). Their cycling performances and discharge–charge curves were determined using a programmable battery cycler (CT-4008-5 V-10 mA, Neware, New Castle, Delaware) at 1.8–2.6 V and at C/20–1C rates with 1C = 1672 mA g^−1^. Cyclic voltammetry (CV) analyses were performed using a potentiostat (VMP-300, Biologic, Grenoble, France) at the aforementioned voltage range and at various scan rates (0.010 to 0.030 mV s^−1^). Electrochemical impedance spectra were recorded using the aforementioned potentiostat from 1 MHz to 10 mHz. The ionic conductivity of polyacrylonitrile films was analyzed from 1 MHz to 100 Hz at various temperatures (from room temperature to 70 °C in increments of 10 °C), and was calculated by *σ* = *d*/*A* × *R_b_*, where *σ*, *d*, *A*, and *R_b_* are the ionic conductivity, thickness, area, and bulk resistance of a polyacrylonitrile film, respectively [44].

## 3. Results and Discussion

### 3.1. Material Characterization of Electrospun Polyacrylonitrile Nanofiber Film

**Figure 1:**
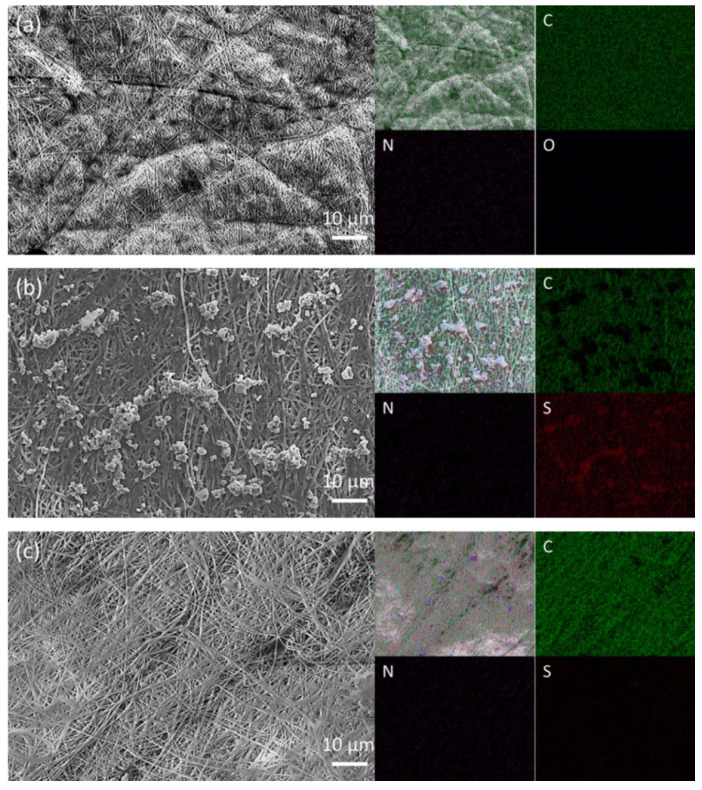
Morphology and element analyses of (**a**) an electrospun polyacrylonitrile nanofiber film, (**b**) a cathode-facing cycled polyacrylonitrile film, and (**c**) an anode-facing cycled polyacrylonitrile film. In the elemental mapping results, C represents carbon, N represents nitrogen, and S represents sulfur.

Figure 1a shows the microstructure of a polyacrylonitrile film sample, revealing that it consists of closely packed electrospun fibers and forms non-interwoven electrospun layers. These complex and non-interwoven fibric networks prevent the positive and negative electrodes contacting each other and suppress the migration of liquid polysulfide species [13,19]. This is highlighted by Figure 1b,c, which depict the cathode-facing side and anode-facing side of a polyacrylonitrile film retrieved from a cycled lithium–sulfur cell, respectively. The cycled polyacrylonitrile film from the cathode-facing side was coated with the peel-off active material, and the elemental analysis of this material contained a strong signal for elemental sulfur. In contrast, the cycled polyacrylonitrile film from the anode-facing side had a smooth surface, devoid of precipitated active material, and its elemental analysis contained no signal for elemental sulfur. Similarly, the energy dispersive X-ray spectroscopy analysis (see data in the Supporting Information) of samples from the cathode-facing and anode-facing sides of the cycled polyacrylonitrile film showed strong and weak intensities of sulfur, respectively (Figure S1). Therefore, the microstructural and elemental analyses indicate that the polysulfide cathode is well stabilized by the polyacrylonitrile film for high retention of the active material and has a tight connection with the polyacrylonitrile film for a smooth lithium-ion transfer. At the other side of the cell, the lithium anode is well protected from the corrosion of polysulfides and also has a smooth electrode/electrolyte interface. These positive morphological and elemental features benefit the improvement of the electrochemical retention and rate performance of the lithium-sulfur cell with high reversibility and efficiency [6,7,13].

**Figure 2:**
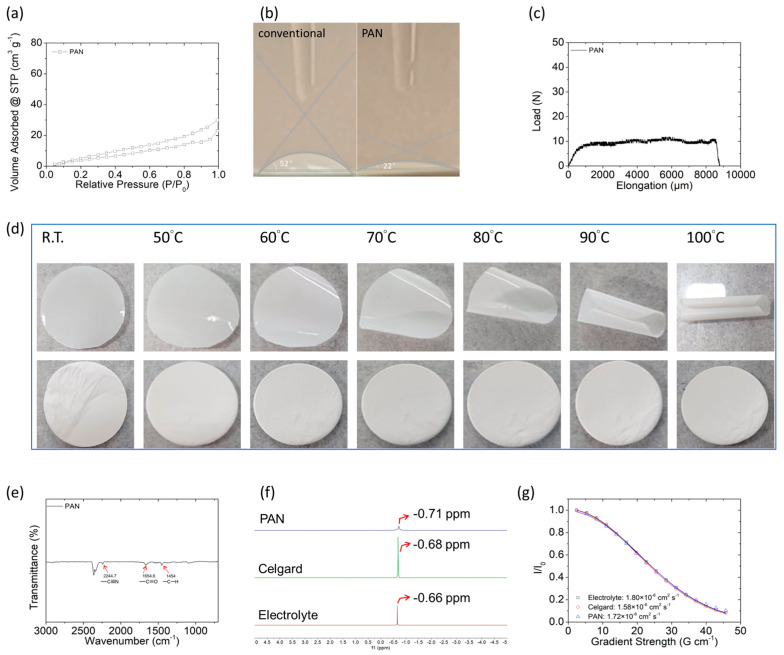
(**a**) Adsorption–desorption isothermal curve of polyacrylonitrile (PAN) film; (**b**) electrolyte affinities of conventional and PAN films; (**c**) elongation test of PAN film; (**d**) heat treatment of conventional polypropylene (upper) and PAN film (lower) separators; (**e**) FTIR analysis of PAN film; (**f**) ^7^Li NMR of electrolyte, conventional separator, and PAN film separator; and (**g**) DOSY calculations of electrolyte and electrolyte-soaked PAN film and conventional polypropylene separator, respectively.

Figure 2 summarizes a series of the key physical and chemical analytical data of the polyacrylonitrile film, which are consistent with the material characteristics necessary to serve as a high-performance separator in the advanced lithium–sulfur cell. Figure 2a shows the nitrogen adsorption–desorption isotherms of the polyacrylonitrile film, confirming the low nanoporosity and low surface area of the electrospun fibers. The total nanopore volume and specific surface area of the polyacrylonitrile film were 0.02–0.03 cm^3^ g^−1^ and 16–23 m^2^ g^−1^, respectively. The closely packed fibric network built up by non-nanoporous electrospun fibers aims to suppress the free diffusion of polysulfides and to maintain the fast penetration of liquid electrolyte. To support the latter feature, Figure 2b shows that the polyacrylonitrile film had a smaller wetting angle (~22°) as compared to that of a commercial separator (~52°), which confirms the high electrolyte affinity and wetting capability of the polyacrylonitrile film. As a result, the polyacrylonitrile film showed a higher electrolyte uptake (277%) and a higher electrolyte retention (65%) after drying for an hour than those obtained by the commercial separator. Thus, Figure 2a,b demonstrate that the non-interwoven electrospun layers consisting of non-nanoporous electrospun fibers enabled good electrolyte penetration and affinity [7,12].

Figure 2c shows that the polyacrylonitrile film underwent substantial elongation (>8000 μm) under a load of 10 N, which attests to the mechanical strength of the electrospun fiber network. Figure 2d and Figure S2 show the results of the heat stability and flammability tests of the polyacrylonitrile film, respectively. In the former heat-stability test, the 200 µm polyacrylonitrile film maintained a flat and unchanged form from room temperature (25 °C) to 100 °C, indicating its good thermostability at high temperatures; in contrast, the conventional separator with a thickness of 25 µm began to twist at 50–60 °C (Figure 2d). In the latter flammability test, the polyacrylonitrile film did not burn, but the commercial separator was burned and thus decomposed within seconds (Figure S2). Therefore, the analyses of mechanical strength, heat stability, and flammability demonstrate the high structural and thermal stability of the polyacrylonitrile film. In addition to the material characteristics, a thick electrospun separator might improve the mechanical properties and the stability and safety performance. This allows the polyacrylonitrile film to offer the electrochemical cell stable and safe high-energy-density performance [1,6,7]. 

Figure 2e shows the FTIR spectrum of the polyacrylonitrile film; the peaks at 2244.7, 1654.6, and 1454 cm^−1^ represent nitrile (C≡N), carbonyl, and alkyl bonds, respectively, confirming the presence of polyacrylonitrile. Figure 2f shows the ^7^Li NMR spectra of the electrolyte, conventional polypropylene separator, and polyacrylonitrile film separator, demonstrating the effect of the highly electronegative C≡N on the chemical shift of the ^7^Li peak (as discussed below). The diffusion-ordered spectroscopy (DOSY) calculation shown in Figure 2g reveals the lithium-ion diffusion coefficients of the electrolyte, conventional polypropylene separator, and polyacrylonitrile film separator, respectively. The chemical shift of the Li peak in the ^7^Li NMR spectrum of the electrolyte-wetted polyacrylonitrile film was upfield from its position in the ^7^Li NMR spectra of the electrolyte and electrolyte-wetted conventional separator. This demonstrated that the polyacrylonitrile film interacted with lithium ions and accounted for its high lithium-ion diffusion coefficient (1.72 × 10^−6^ cm^2^ s^−1^), which is similar to that of the liquid electrolyte (1.80 × 10^−6^ cm^2^ s^−1^) but higher than that of electrolyte-wetted conventional polypropylene separator (1.58 × 10^−6^ cm^2^ s^−1^). This indicates that the thick polyacrylonitrile film would not block the penetration of electrolyte and slow down the transfer of lithium ions. As a result, the highly safe polyacrylonitrile film has excellent lithium-ion transfer capability for the high-performance conversion lithium–sulfur battery chemistry [7,10,11,12].

Overall, the abovementioned material analyses show that the polyacrylonitrile film features complex non-interwoven fibric networks with a layered structure consisting of non-nanoporous fibers. This indicates that the polyacrylonitrile film is well penetrated by electrolytes and thus can enable the high diffusion of lithium ions, while also exhibiting good mechanical strength, thermostability, and low flammability.

### 3.2. Electrochemical Performance of Electrospun Polyacrylonitrile Nanofiber Film

**Figure 3:**
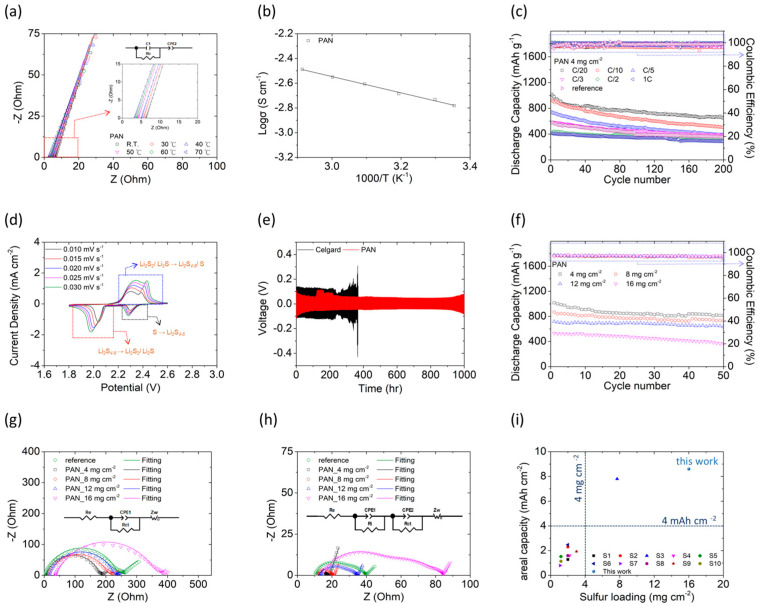
(**a**) Ionic conductivity and (**b**) Arrhenius plot of polyacrylonitrile (PAN) film; (**c**) cyclability of lithium–sulfur cells with different separators; (**d**) CV analysis of lithium–sulfur cells with PAN film; (**e**) lithium//lithium symmetrical cells with different separators; (**f**) cyclability, (**g**) uncycled impedance analysis, and (**h**) cycled impedance analysis of lithium–sulfur cells with increasingly high sulfur loadings; (**i**) electrochemical performances of lithium–sulfur cells containing various electrospun separators.

Figure 3a,b show that from room temperature (25 °C) to 70 °C, the polyacrylonitrile film exhibited the high ionic conductivities (1.65–3.26 mS cm^−1^) [44] necessary for the lithium–sulfur battery cathode to ensure excellent long-term cycle stability and promising high-loading capability. Figure 3c shows the long-term cyclabilities, with consideration of rate performance from slow C/20 rate to fast 1C rate, of a high-loading polysulfide cathode (4 mg cm^−2^) equipped with a polyacrylonitrile film separator and a conventional separator at C/10 rate as a reference, respectively. The resulting lithium–sulfur cell using the polyacrylonitrile film revealed high peak discharge capacities of 929 mA·h g^−1^ when cycled at C/10. At the same cycling rate, the reference cell with the commercial polypropylene separator showed a decrease in charge-storage capacity by 160 mA·h g^−1^ and had only 769 mA·h g^−1^. The polyacrylonitrile film and the commercial polypropylene separator showed capacity retention of approaching 60% and only 25% after 200 cycles, respectively. These demonstrate the significant improvement brought about by the use of the polyacrylonitrile film in the electrochemical utilization and stability of lithium–sulfur cells. In addition to the improved charge-storage and reversible capacities of cells containing a polyacrylonitrile film, those that also contained a high-loading polysulfide cathode with 4 mg cm^−2^ sulfur exhibited high discharge capacities of 1017, 737, 587, 422, and 411 mA·h g^−1^ when cycled at C/20, C/5, C/3, C/2, and 1C, respectively. After 200 cycles, the cells equipped with a polyacrylonitrile film retained excellent discharge–charge efficiencies (approaching 99%) and high capacity retentions (50–80%). The long cycle life of these polyacrylonitrile film-equipped cells at various current densities indicates the excellent rate performance, fast reaction kinetics, and stable electrochemical reactions of these cells [6,7,18].

Figures S3–S8 show the discharge–charge voltage profiles of cells equipped with our polyacrylonitrile film and cycled at C/20 to 1C. The cells displayed complete upper and lower discharge plateaus at cell discharge and a continuous charge plateau at cell charge when cycled at different rates for 200 cycles, indicating their good reaction capability and reversibility. Specifically, the discharge reaction of the cell initiates from the upper discharge plateau, which represents the reduction conversion from solid-state sulfur to liquid-state lithium polysulfides. Subsequently, the lower discharge plateau represents the reduction reaction of polysulfides to solid-state lithium sulfides. During the charge state, the charge plateau belongs to the oxidation reaction from sulfides to polysulfides and sulfur [6,7,8]. The remaining complete discharge and charge curves overlapped during the long cycle life of the cell, showing its excellent electrochemical stability involving high polysulfide retention and high conversion of sulfides [10,33]. Figure S9 shows the hysteresis between the charge and discharge processes in a cell equipped with the polyacrylonitrile film, revealing its low polarization when cycled at C/20 to 1C and slightly higher polarization when cycled at C/2 and 1C. Although the sluggish conversion nature of the large amount of sulfur in the cathode, as was visible at high-rate testing, led to a relatively high polarization of the cells cycled at C/2 and 1C, the high-loading polysulfide cathode maintained a stable cycling performance for a long life. These demonstrate that the polyacrylonitrile film could enable the high-loading polysulfide cathode to achieve smooth lithium-ion diffusion and have low impedance. Moreover, it blocked the diffusion of polysulfides and thus prevented uncontrollable deposition of the solid-state insulating material onto the electrode.

Figure 3d shows the cyclic voltammetry analyses of a polyacrylonitrile film-equipped cell at increasing scan rates: 0.010, 0.015, 0.020, 0.025, and 0.030 mV s^−1^. As the scan rate increased, the same redox reactions were maintained in the cell, which also exhibited almost unchanged discharge–charge redox peaks. The chemical reaction of the polysulfide cathode during the discharge process showed two cathodic peaks, which show the reduction conversion of sulfur to polysulfides (black box: S → Li_2_S_4–8_) and of polysulfides to sulfides (red box: Li_2_S_4–8_ → Li_2_S_2_/Li_2_S). The chemical reaction of the cathode during the charge process displayed two anodic peaks, which represent the charge conversion of sulfides to polysulfides and sulfur (blue box: Li_2_S_2_/Li_2_S → Li_2_S_4–8_/S) [6,7,8]. At each scan rate, the cell was analyzed three times and overlapping CV curves were obtained; the unshifted cathodic and anodic peaks are shown in Figures S10–S14. As a result, the obtained CV data affirm the excellent electrochemical reversibility and stability as well as the promising reaction kinetics of the cell with the polyacrylonitrile film. The peak current and the scan rates of the cell were collected to determine the lithium-ion diffusion behavior of the polyacrylonitrile film. The results in Figure S15 were obtained using the Randles–Ševčík equation and indicate that the film had high lithium-ion diffusion coefficients, ranging from 6.11 × 10^−8^ to 9.28 × 10^−9^ cm^2^ s^−1^ [6,45]. Overall, the CV analysis demonstrates that the polyacrylonitrile film improves the electrochemical stability and reaction capabilities of a lithium–sulfur cell containing a high amount of active material by inhibiting the rapid loss of polysulfide, facilitating lithium-ion transfer, and maintaining stable electrochemical redox reactions. 

Figure 3e shows the lithium plating and stripping behavior of symmetrical lithium cells equipped with a polyacrylonitrile film and a commercial separator at a current density of 1 mA cm^−2^. The cell equipped with a polyacrylonitrile film exhibited stable and smooth lithium plating and stripping behavior for 1000 h, which was significantly longer (indicative of greater lithium-metal stability) than that of the cell equipped with a commercial separator (368 h). Moreover, after 1000 h of testing, the symmetrical lithium cell equipped with the polyacrylonitrile film remained stable with no apparent short circuit. The lithium-metal electrodes were subsequently retrieved from these cycled symmetrical lithium cells and examined. Figure S16 indicates that the electrode from the cell equipped with the commercial separator had an irregular surface due to the presence of lithium dendrites. However, the electrode from the cell equipped with the polyacrylamide film had a smooth surface, demonstrating the close electrochemical connection that was achieved in this cell between the electrode and the electrolyte. Taken together, the results from the above-described electrochemical and morphological analysis confirm that cells containing a polyacrylonitrile film have highly stable stripping and plating behaviors and well-protected anodes [10,11,12,13].

Furthermore, the polyacrylonitrile film enabled excellent active-material utilization and efficiency at the polysulfide cathode and high electrochemical stability at the lithium-metal electrode in cells. Figure 3f–i demonstrate the feasibility of adopting the polyacrylonitrile film to develop a high-loading polysulfide cathode in cells for high specific capacity and energy density. Figure 3f shows the cyclability of high-loaded polysulfide cathodes (with high sulfur loadings of 4, 8, 12, and 16 mg cm^−2^) and high sulfur contents (50, 68, 75, and 80 wt%). The polyacrylonitrile film enabled the high-loading cathodes with sulfur loadings of 4, 8, 12, and 16 mg cm^−2^ to attain high discharge capacities of 1017, 867, 718, and 533 mA·h g^−1^ at C/20, respectively. Figures S17–S20 illustrate that the high-loading cathodes retained the same complete charge and discharge plateaus as their amount of sulfur and their cycle life increased. Moreover, the discharge and charge curves of these cathodes overlapped and illustrate that these cathodes maintained a low polarization (Figure S21). These electrochemical characteristics demonstrate the high electrochemical utilization, efficiency, and stability of these high-loading polysulfide cathodes, which were due to the presence of the polyacrylonitrile film and resulted in these cathodes having high areal capacities (4.1–8.6 mA·h cm^−2^) and high energy densities (8.6–18.1 mW·h cm^−2^). Figure 3g,h compare the before-and-after-cycling electrochemical impedances of the cathodes in cells equipped with the polyacrylonitrile film. *R_e_*, *R_ct_*, *R_i_*, and *Z_w_* represent the cathodes’ bulk resistance, charge-transfer resistance, interface resistance, and lithium-ion diffusion Warburg impedance, respectively [46,47,48]. The uncycled high-loading cathodes with 4 (50 wt%), 8 (68 wt%), 12 (75 wt%), and 16 (80 wt%) mg cm^−2^ sulfur showed *R_ct_* values of 170.3, 187.3, 208.1, and 321.3 Ω, respectively. However, compared with the corresponding uncycled high-loading cathode, the reference cathode with 4 (50 wt%) mg cm^−2^ sulfur showed a much higher *R_ct_* value (238.2 Ω). After cycling, the relocation of active material in the cathode region of the cells protected with a polyacrylonitrile film resulted in a decrease in their *R_ct_* values to 5.8, 6.4, 15.5, and 40.8 Ω, respectively. Moreover, the smooth interface between the electrode and electrolyte in these cells led to *R_i_* values of only 1.1, 3.7, 5.0, and 28.6 Ω, respectively; in contrast, the reference cell showed high resistances (14.4 Ω (*R_ct_*) and 14.6 Ω (*R_i_*)). The consistently low impedances of the cells equipped with the polyacrylonitrile film demonstrate that the film enhanced the electrochemical reaction and, crucially, maintained this good redox-reaction capability as the sulfur loading increased and after cell operation. Figure 3i and Table S1 indicate the feasibility of our work and the research trends in the use of electrospun films as separators in lithium–sulfur batteries. As our polyacrylonitrile film was developed to address the main problem in this area—the high resistance of high-loading sulfur cathodes [6,7,8,9,10,11,12,13,47]—it allowed the application of a high-loading (16 mg cm^−2^) polysulfide cathode containing 80 wt% sulfur, which maintained good electrochemical utilization for achieving the high areal specific capacity (8.6 mA·h cm^−2^) and energy density (18.1 mW·h cm^−2^). These cell-design parameters and the electrochemical performance exceed the requirements for the electric vehicle power source (i.e., 2–4 mA∙h cm^−2^) and commercial cathode (i.e., 10–15 mW∙h cm^−2^) [6,7,8,9,10,11,12,37,38,39,40].

## 4. Conclusions

In this study, we electrospun a polyacrylonitrile nanofiber film and used it as a separator in a lithium–sulfur cell. The polyacrylonitrile film had high mechanical strength and high electrolyte affinity but low nanoporosity. It endowed a high-loading polysulfide cathode with excellent rate performance from C/20 to 1C and a long cycle life (200 cycles). The presence of the polyacrylonitrile film led to a lithium-metal electrode exhibiting stable lithium stripping and plating reactivity for 1000 h. The stabilized polysulfide cathode and lithium electrode generated by the use of the polyacrylonitrile film enabled a lithium–sulfur cell to have high sulfur loadings and contents (4, 8, 12, and 16 mg cm^−2^ and 50, 68, 75, and 80 wt%). Moreover, the polyacrylonitrile film stabilized a high-loading polysulfide cathode, such that it achieved an impressively high areal capacity and high energy density (8.6 mA·h cm^−2^ and 18.1 mW·h cm^−2^, respectively).

## Supplementary Materials

The following are available online at https://www.mdpi.com/article/10.3390/polym15061460/s1, Figure S1: Energy-dispersive X-ray spectroscopy elemental analysis, Figure S2: Flammability testing, Figures S3–S8: Discharge–charge voltage profiles, Figure S9: Hysteresis between charge and discharge processes, Figures S10–S14: Cyclic voltammetry analysis, Figure S15: Lithium-ion diffusion coefficient profiles, Figure S16: Morphology of a lithium-metal electrode, Figure S17–S20: Discharge–charge voltage profiles, Figure S21: Hysteresis between charge and discharge processes, Table S1: Analysis of the sulfur cathodes with the electrospun separators reported recently, Supporting References: [34,42,49,50,51,52,53,54,55,56].
