# Supplementary material for: Electrochemically Stable Rechargeable Lithium–Sulfur Batteries Equipped with an Electrospun Polyacrylonitrile Nanofiber Film
Source: Polymers (Basel). 2023 Mar 15;15(6):1460. doi: 10.3390/polym15061460 (PMC10057069; doi:10.3390/polym15061460)
Supplement: Supplementary file 1 [file polymers-15-01460-s001.zip › polymers-2226280-supplementary.pdf]

Supporting Figures

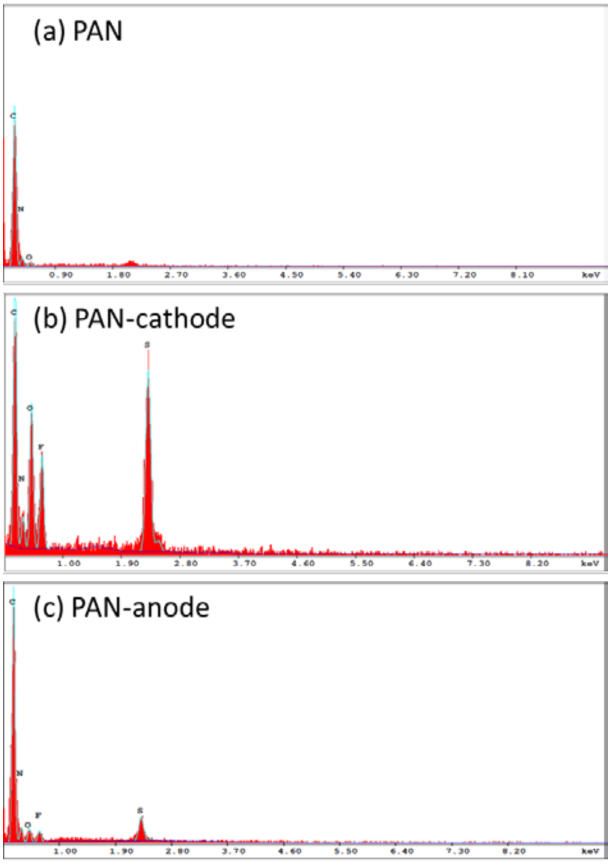

Figure S1. Energy-dispersive X-ray spectroscopy elemental analysis of (a) an electrospun polyacrylonitrile (PAN) nanofiber film, (b) a cathode-facing cycled polyacrylonitrile film, and (c) an anode-facing cycled polyacrylonitrile film.

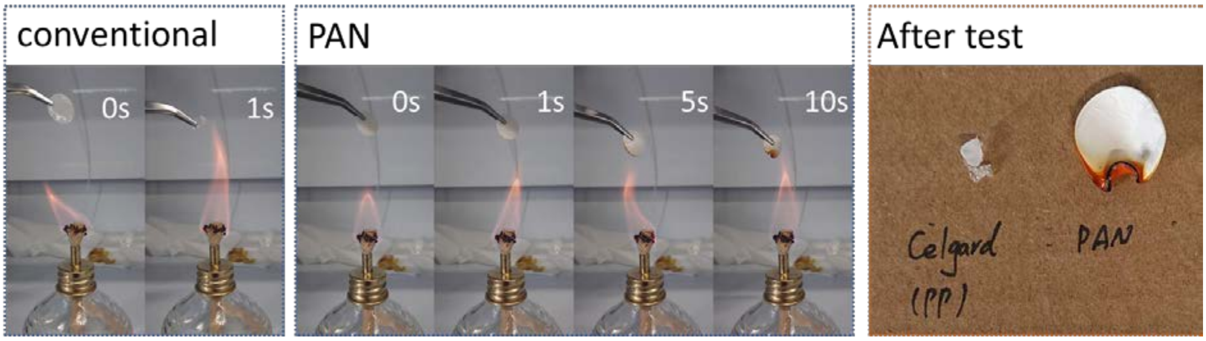

Figure S2. Flammability testing of conventional (polypropylene (PP)) and polyacrylonitrile (PAN) film separators.

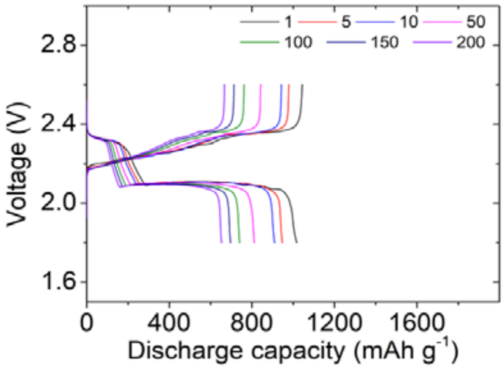

Figure S3. Discharge–charge voltage profiles of a lithium–sulfur cell equipped with a polyacrylonitrile film and cycled at C/20.

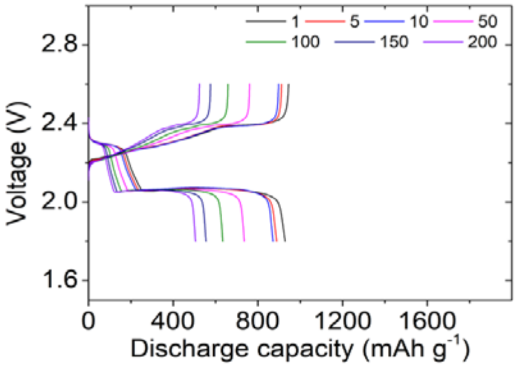

Figure S4. Discharge–charge voltage profiles of a lithium–sulfur cell equipped with a polyacrylonitrile film and cycled at C/10.

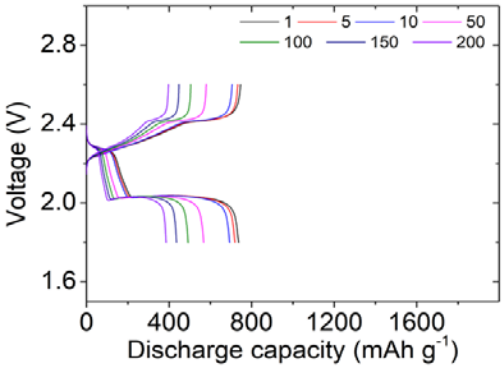

Figure S5. Discharge–charge voltage profiles of a lithium–sulfur cell equipped with a polyacrylonitrile film and cycled at C/5.

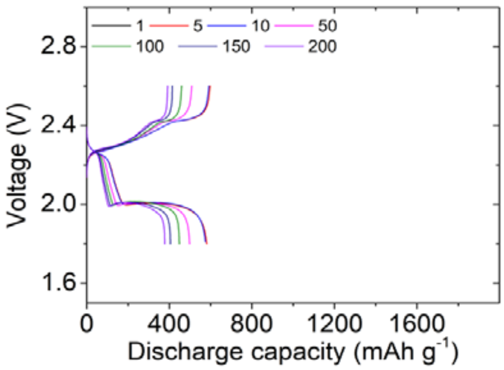

Figure S6. Discharge–charge voltage profiles of a lithium–sulfur cell equipped with a polyacrylonitrile film and cycled at C/3.

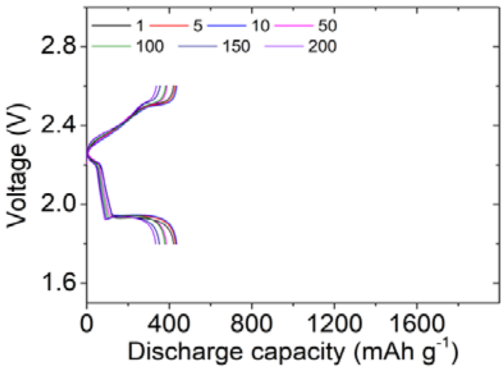

Figure S7. Discharge–charge voltage profiles of a lithium–sulfur cell equipped with a polyacrylonitrile film and cycled at C/2.

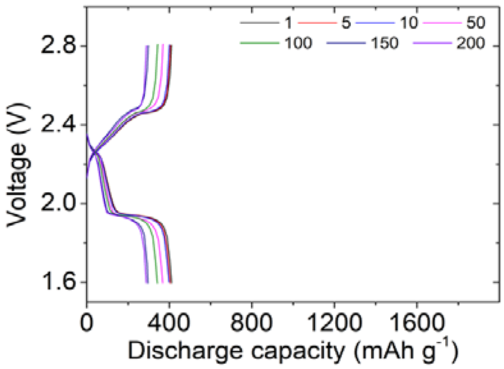

Figure S8. Discharge–charge voltage profiles of a lithium–sulfur cell equipped with a polyacrylonitrile film and cycled at 1C.

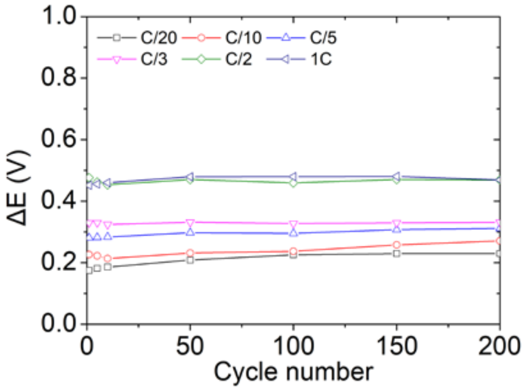

Figure S9. Hysteresis between charge and discharge processes in a lithium–sulfur cell equipped with a polyacrylonitrile film at various cycling rates for 200 cycles.

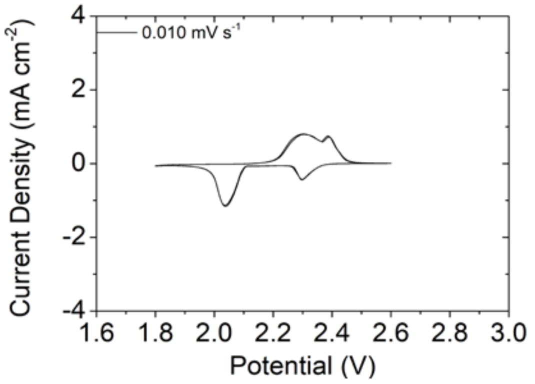

Figure S10. Cyclic voltammetry analysis at 0.010 mV s^{-1} of a lithium–sulfur cell equipped with a polyacrylonitrile film.

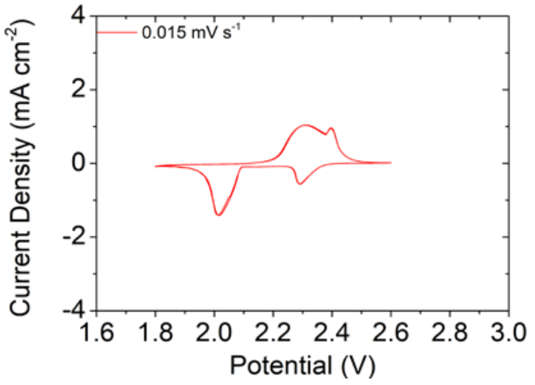

Figure S11. Cyclic voltammetry analysis at 0.015 mV s^{-1} of a lithium–sulfur cell equipped with a polyacrylonitrile film.

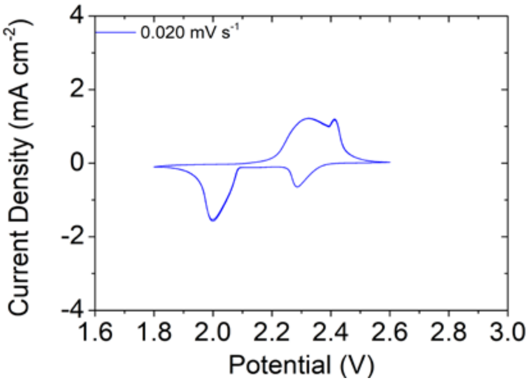

Figure S12. Cyclic voltammetry analysis at 0.020 mV s^{-1} of lithium–sulfur cell equipped with a polyacrylonitrile film.

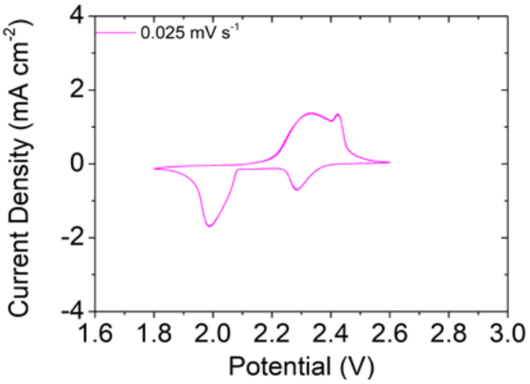

Figure S13. Cyclic voltammetry analysis at 0.025 mV s^{-1} of a lithium–sulfur cell equipped with a polyacrylonitrile film.

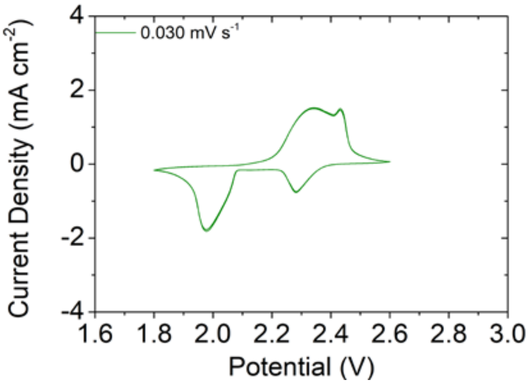

Figure S14. Cyclic voltammetry analysis at 0.030 mV s^{-1} of a lithium–sulfur cell equipped with a polyacrylonitrile film.

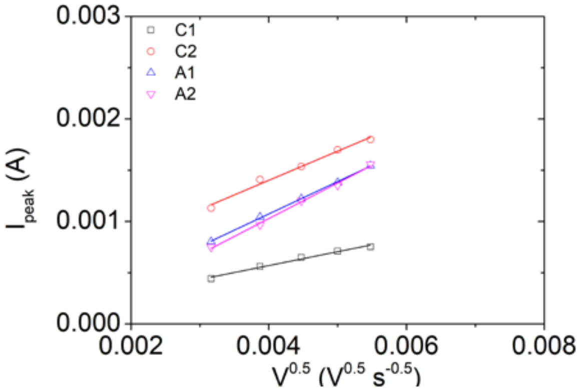

Figure S15. Lithium-ion diffusion coefficient profiles of the cyclic voltammetry analysis at various rates using a lithium–sulfur cell equipped with a polyacrylonitrile film.

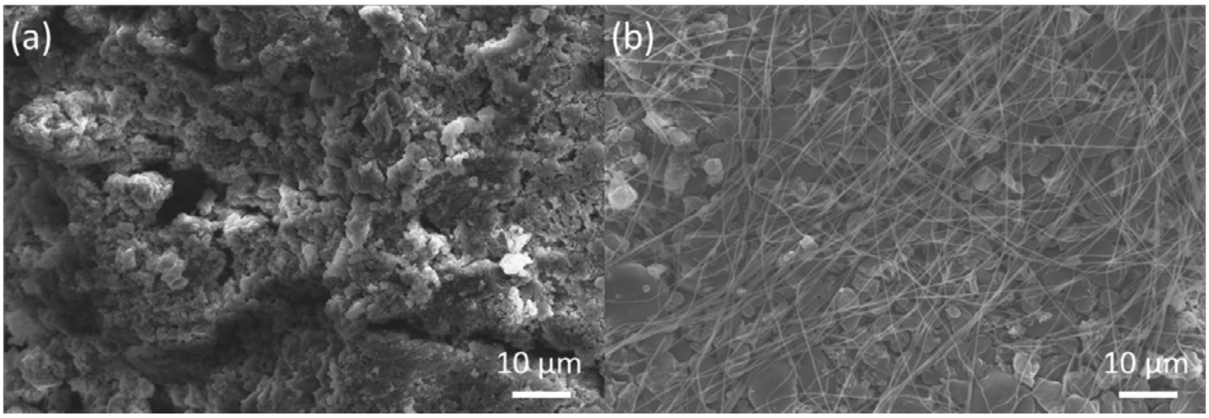

Figure S16. Morphology of a lithium-metal electrode after undergoing stripping and plating reactions from a cell equipped with (a) a conventional separator or (b) a polyacrylonitrile film separator.

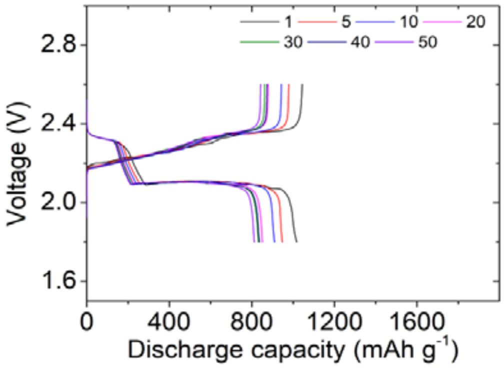

Figure S17. Discharge–charge voltage profiles of a lithium–sulfur cell (with a sulfur loading and content of 4 mg cm^{-2} and 50 wt%, respectively) equipped with a polyacrylonitrile film.

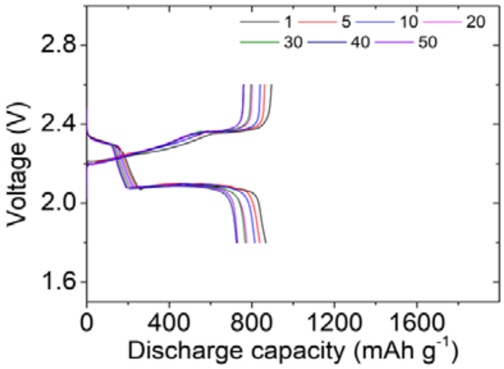

Figure S18. Discharge–charge voltage profiles of a lithium–sulfur cell (with a sulfur loading and content of 8 mg cm^{-2} and 68 wt%, respectively) equipped with a polyacrylonitrile film.

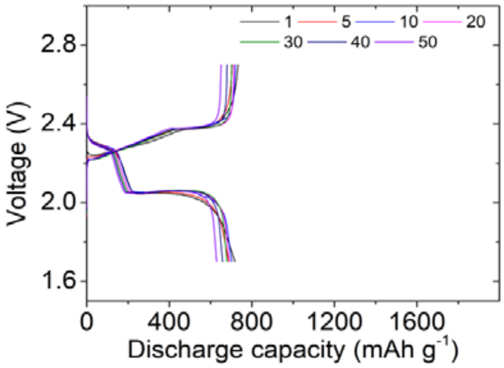

Figure S19. Discharge–charge voltage profiles of a lithium–sulfur cell (with a sulfur loading and content of 12 mg cm^{-2} and 75 wt%, respectively) equipped with a polyacrylonitrile film.

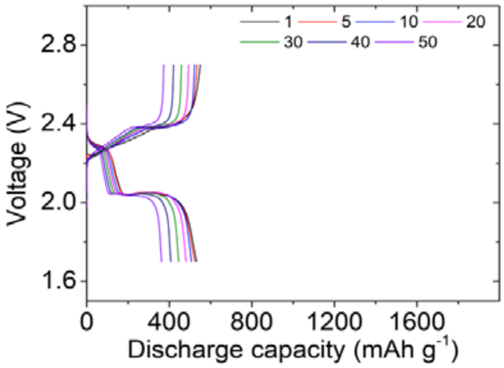

Figure S20. Discharge–charge voltage profile of a lithium–sulfur cell (with a sulfur loading and content of 16 mg cm^{-2} and 80 wt%, respectively) equipped with a polyacrylonitrile film.

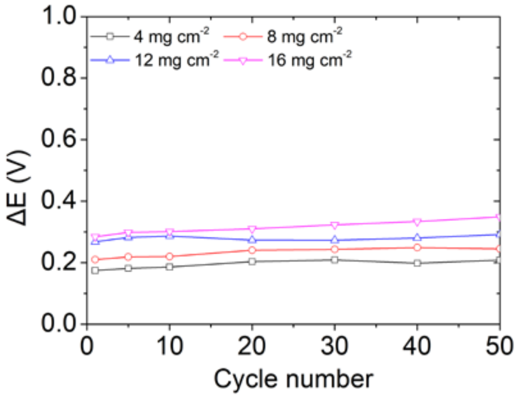

Figure S21. Hysteresis between charge and discharge processes in high-sulfur-loading lithium–sulfur cells equipped with a polyacrylonitrile film.

Supporting Tables

Table S1. Analysis of the sulfur cathodes with the electrospun separators reported recently.

	A	B	C	D	E	F
[49]	S/KB	60	2	PAN/PAA	1.3	2.7
[34]	S/KB	70	2	PAN–PDAAQ	2.3	4.8
[50]	rGO-S	N/A	7.7	T@LPP	7.8	16.4
[51]	S/C	N/A	2.2	PEI/PAN	1.6	3.4
[52]	S/CNF	N/A	1.2	F-Cu-BTC-PMIA	1.5	3.2
[53]	S/C	60	2	CPP@PVA/ZIF-8	2.5	5.2
[54]	C@S	73	1.1	T@LPP	0.8	1.7
[42]	S	60	2	C-PVA/PAA-Li	1.6	3.4
[55]	S/C	N/A	3	ZIF@T-PVDF	1.9	4.1
[56]	S/MWCNT	53	1.2	PHB/Co-CNF	1.14	2.4
Our work	pristine	80	16	PAN	8.6	18.1

A: Cathode, B: sulfur content [wt%], C: sulfur loading [mg cm^{-2}], D: Electrospinning separator, E: areal capacity [mA·h cm^{-2}], F: energy density [mW·h cm^{-2}].

Supporting References

[49] Zhu, X.; Ouyang, Y.; Chen, J.: Zhu, X.; Luo, X.; Lai, F.; Zhang, H.; Miao, Y.-E.; Liu, T. *In Situ* Extracted Poly(acrylic acid) Contributing to Electrospun Nanofiber Separators with Precisely Tuned Pore Structures for Ultra-stable Lithium–Sulfur Batteries. *J. Mater. Chem. A* **2019**, *7*, 3253–3263.

[34] Kiai, M.S.; Eroglu, O.; Kizil, H. Electrospun Nanofiber Polyacrylonitrile Coated Separators to Suppress the Shuttle Effect for Long-life Lithium–Sulfur Battery. *J. Appl. Polym. Sci*. **2019**, *137*, 48606.

[50] Zhou, C.; He, Q.; Li, Z.; Meng, J.; Hong, X.; Li, Y.; Zhao, Y.; Xu, X.; Mai, L. A Robust Electrospun Separator Modified with In Situ Grown Metal-organic Frameworks for Lithium-Sulfur Batteries. *Chem. Eng. J*. **2020**, *395*, 124979.

[51] Hu, M.; Ma, Q.; Yuan, Y.; Pan, Y.; Chen, M.; Zhang, Y.; Long, D. Grafting Polyethyleneimine on Electrospun Nanofiber Separator to Stabilize Lithium Metal Anode for Lithium Sulfur Batteries. *Chem. Eng. J*. **2020**, *388*, 124258.

[52] Deng, N.; Wang, L.; Feng, Y.; Liu, M.; Li, Q.; Wang, G.; Zhang, L.; Kang, W.; Cheng, B.; Liu, Y. Co-based and Cu-based MOFs Modified Separators to Strengthen the Kinetics of Redox Reaction and inhibit Lithium-dendrite for Long-life Lithium-Sulfur Batteries. *Chem. Eng. J*. **2020**, *388*, 124241.

[53] Zheng, S.; Zhu, X.; Ouyang, Y.; Chen, K.; Chen, A.-L.; Fan, X.; Miao, Y.-E; Liu, T.; Xie, Y. Metal–Organic Framework Decorated Polymer Nanofiber Composite Separator for Physiochemically Shielding Polysulfides in Stable Lithium–Sulfur Batteries. *Energy Fuels* **2021**, *35*, 19154–19163.

[54] Lan, F.; Zhang, H.; Fan, J.; Xu, Q.; Li, H.; Min, Y. Electrospun Polymer Nanofibers with TiO_2@NiCo-LDH as Efficient Polysulfide Barriers for Wide-Temperature-Range Li–S Batteries. *ACS Appl. Mater. Interfaces* **2021**, *13*, 2734–2744.

[42] Zhou, C.; Wang, J.; Zhu, X.; Chen, K.; Ouyang, Y.; Wu, Y.; Miao, Y.-E; Liu, T. A Dual-functional Poly(vinyl alcohol)/Poly(lithium acrylate) Composite Nanofiber Separator for Ionic Shielding of Polysulfides Enables High-rate and Ultra-stable Li-S Batteries. *Nano Research* **2021**, *14*, 1541-1550.

[55] Fang, Y.; Wang, G.; Kang, W.; Deng, N.; Cheng, B. Taming Polysulfides and Facilitating Lithium-ion Migration: Novel Electrospinning MOFs@PVDF-based Composite Separator with Spiderweb-like Structure for Li-S Batteries. *Electrochim. Acta* **2021**, *365*, 137344.

[56] Liang, X.; Wang, L.; Wang, Y.; Liu, Y.; Sun, Y.; Xiang, H. Constructing Multi-functional Composite Separator of PVDF-HFP/h-BN Supported Co-CNF Membrane for Lithium–Sulfur Batteries. *Sustain. Energy Fuel*s **2022**, *6*, 440–448.
